# The Purine Salvage Pathway and the Restoration of Cerebral ATP: Implications for Brain Slice Physiology and Brain Injury

**DOI:** 10.1007/s11064-017-2386-6

**Published:** 2017-08-24

**Authors:** Bruno G. Frenguelli

**Affiliations:** School of Life Science, University of Warwick, Coventry, CV4 7AL UK

**Keywords:** ATP, Adenosine, Stroke, TBI, Purines, Traumatic brain injury

## Abstract

Brain slices have been the workhorse for many neuroscience labs since the pioneering work of Henry McIlwain in the 1950s. Their utility is undisputed and their acceptance as appropriate models for the central nervous system is widespread, if not universal. However, the skeleton in the closet is that ATP levels in brain slices are lower than those found in vivo, which may have important implications for cellular physiology and plasticity. Far from this being a disadvantage, the ATP-impoverished slice can serve as a useful and experimentally-tractable surrogate for the injured brain, which experiences similar depletion of cellular ATP. We have shown that the restoration of cellular ATP in brain slices to in vivo values is possible with a simple combination of d-ribose and adenine (RibAde), two substrates for ATP synthesis. Restoration of ATP in slices to physiological levels has implications for synaptic transmission and plasticity, whilst in the injured brain in vivo RibAde shows encouraging positive results. Given that ribose, adenine, and a third compound, allopurinol, are all separately in use in man, their combined application after acute brain injury, in accelerating ATP synthesis and increasing the reservoir of the neuroprotective metabolite, adenosine, may help reduce the morbidity associated with stroke and traumatic brain injury.

## Introduction

### Brain Slices: We Have a Problem

**Fig. 1:**
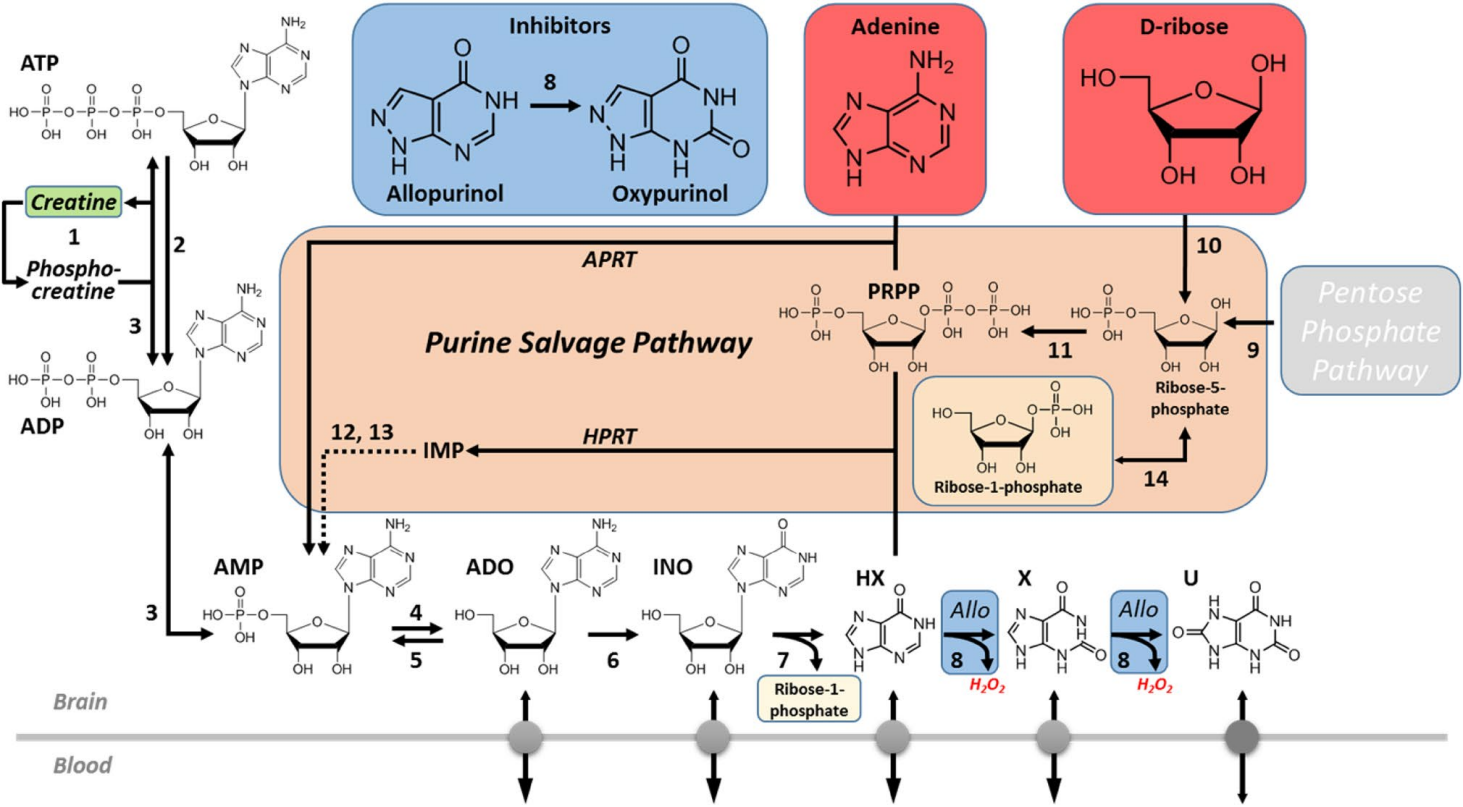
Adenine nucleotide metabolism and synthesis via the purine salvage pathway. ATP breakdown results in the production of adenosine (ADO) and other metabolites that can be lost from the brain via equilibrative nucleoside transporters (*light grey spheres*) into the blood. The purine salvage pathway restores adenine nucleotide levels via HPRT (hypoxanthine to IMP) and APRT (adenine to AMP). These reactions require PRPP, a product of the pentose phosphate pathway from which ribose-5-phosphate emerges. Ribose-5-phosphate can be formed directly from d-ribose by ribokinase (10), or via isomerisation of inosine (INO)-derived ribose-1-phosphate (14), thereby increasing PRPP levels. Adenine and d-ribose feed into the salvage pathway and increase tissue ATP levels via adenylate kinase-mediated phosphorylation of AMP and thence to ATP (3). AMP can also be formed from adenosine via adenosine kinase (5). Allopurinol (Allo), following its conversion to oxypurinol (8), prevents the breakdown of hypoxanthine (HX) to xanthine (X), and from xanthine to uric acid (U). Uric acid has its own bidirectional transporter (*dark grey* sphere). Creatine can buffer the decline in ATP levels via creatine kinase-catalysed substrate-level phosphorylation of ADP (1). *1* creatine kinase; *2* ATPases; *3* adenylate kinases; *4* 5′nucleotidase; *5* adenosine kinase; *6* adenosine deaminase; *7* purine nucleoside phosphorylase; *8* xanthine oxidase; *9* ribulose 5-phosphate isomerase; *10* ribokinase; *11* phosphoribosylpyrophosphate synthetase; *12* adenylosuccinate synthetase; *13* adenylosuccinate lyase; *14* phosphopentomutase; *APRT* adenine phosphoribosyltransferase; *HPRT* hypoxanthine–guanine phosphoribosyltransferase; *PRPP* phosphoribosyl pyrophosphate. Adenine/d-ribose (*red*), creatine (*green*) and allopurinol (*blue*) are colour-coded as they appear in subsequent figures. (Color figure online)

In the 1950s Henry McIlwain popularised the use of surgically isolated brain slices as a means to study in vitro the biochemical and electrical activity of the mammalian brain [1]. Whilst this was, and remains, an extremely valuable model system, it was clear to McIlwain that the brain slice was biochemically very different from the intact brain [2–7]. This appreciation had come from extensive studies in his own lab, but also from others, that had shown very rapid post mortem changes in the brains of animals, not least of which in ATP and phosphocreatine, which, by donating a phosphate to ADP, delays the loss of ATP (Fig. 1) [8, 9]. This rapidity of decline of ATP and phosphocreatine was understood at the time to reflect the high metabolic rate of the brain [2]. We now know that the majority of cerebral metabolism is directed primarily towards supporting synaptic transmission [10], and which explains the ATP-sparing influence of suppressing neuronal activity with anaesthesia during cerebral ischemia [11].

Whilst phosphocreatine levels could be returned close to values reported in vivo by inclusion of creatine in the incubation medium [4], and which provided reassurance as to the biochemical viability of brain slices and the general validity of the slice approach, restoring ATP levels provided to be more of a challenge. This challenge has persisted, not helped by the steep oxygen gradients found in brain slices [12–14] that may exacerbate the initial insult, hamper metabolic restitution, and indeed confound experimental observations [15, 16]. Thus, for the most part, ATP in brain slices remains at approximately 50% of the levels found in vivo. This has led to the uncharitable description of brain slices as “half dead”, but there is some (metabolic) truth in Konstantin Hossmann’s assertion that brain slices reflect a brain in a post-ischemic, post-traumatic state [17].

### ATP: More Than Just Currency

It should go without saying that the ATP content of brain slices is important: ATP is the primary energy currency of all cells; it powers the Na^+^/K^+^ ATPase that is responsible for the maintenance of the resting membrane potential and the restoration of ionic homeostasis after action potentials and synaptic activity, and which alone accounts for the majority of the brain’s energy budget [10, 18]. ATP critically regulates the ability of kinases to phosphorylate substrates, and is required for protein synthesis, both of which are important for commonly studied slice phenomena such as long-term potentiation (LTP) [19]. ATP also has signalling properties in its own right through at least seven ionotropic (P2X_1−7_) and eight G protein-coupled receptors (GPCRs; P2Y_1,2,4,6,11,12,13,14_), and indirectly through its metabolism to ADP (P2Y_1,12,13_ agonist) and adenosine, which has pronounced actions on the CNS through its four GPCRs (A_1_, A_2A_, A_2B_, A_3_) [20]. Moreover, the ratio of ATP:AMP critically regulates the activity of AMP-activated protein kinase (AMPK), an enzyme that is known to regulate a number of ion channels and which is increasingly being implicated in a host of CNS functions [21, 22]. Importantly for slice physiologists, the activity of AMPK is greater in slices incubated at room temperature (22 °C) compared to ~34 °C [23]. This increased activity was associated with a lower ATP:AMP ratio at room temperature, likely due to the temperature-dependence of adenylate kinase, which interconverts ATP, ADP and AMP (Fig. 1), and which, like many enzymes, operates optimally at physiological temperatures.

### Basis for ATP Depletion

Early attempts to restore ATP levels in brain slices were made by a colleague of McIlwain’s; brain slices (guinea pig cerebral cortex) fixed immediately upon preparation had adenine nucleotides at ~55% of the value of brain tissue frozen in situ; slices incubated in saline fared worse, with adenine nucleotides at ~30% of the in vivo value [24]. This value could be improved with the provision of adenine, inosine and, better still, adenosine in the incubation saline, but still only to ~34–40% of the in situ value, with additional gains (~10%) if a cocktail of adenosine, guanine and creatine were included. A similar approach some 40 years later using adenine and adenosine yielded comparable observations, but with some neuroprotection after oxygen/glucose deprivation (OGD) afforded by adenosine [25].

Adenosine, however, is not a practical adjunct to brain slice incubation solutions given its profound actions on the CNS through its four receptors, some of which provided the neuroprotection described above [26, 27]. In the context of brain slice electrophysiology these actions include: adenosine-mediated internalisation of the inhibitory adenosine A_1_ and A_3_ receptors, but not the excitatory A_2A_ receptor, inhibition of neurotransmitter release, in particular of glutamate, and endocytosis of glutamate AMPA receptors [28–32]. These effects would dramatically shift the balance of excitation and inhibition in the slice preparation and be far removed from the in vivo situation where extracellular levels of adenosine are kept at a very low levels (typically sub-micromolar [33]). This low basal tone of adenosine occurs primarily via the actions of adenosine kinase, which maintains an inward gradient for adenosine through its conversion to AMP (Fig. 1); pharmacological or genetic manipulations of adenosine kinase have pronounced effects on extracellular adenosine and synaptic transmission [34, 35], and its upregulation in the gliosis associated with brain injury contributes to seizure activity and post-traumatic epileptogenesis [36].

Adenine, however, does have potential as an adjunct to slice incubation solutions, but not on its own. This appreciation originally stemmed from observations in the heart in which periods of anoxia or ischemia were accompanied by decreases in myocardial ATP which failed to recover upon reperfusion [37]. Various explanations were proposed, including dysfunction of mitochondria, and lactic acid and pH changes influencing ATP synthesising enzyme kinetics. However, one observation suggested that the basis of this prolonged post anoxic/ischemic ATP depletion was via the loss of ATP precursors from the heart and into the perfusate or blood stream [38] (similar observations were being made by Robert Berne around the same time [39]). The importance of this loss of precursors, which also occurs in man [40], stems from the fact that the heart predominately utilises the purine salvage pathway (Fig. 1) for the resynthesis of adenine nucleotides [39]. Thus, the loss of these precursors is a plausible explanation for the protracted depletion of cardiac ATP after an ischemic episode.

### Myocardium as a Model for ATP Replenishment

Attempts to exploit the purine salvage pathway to restore cardiac adenine nucleotides avoided adenosine, since its profound actions on the cardiovascular system had been known since the 1920s [41], and inosine, since it too was found to exert a direct influence on the heart [42]. Instead, these repletion studies, mainly led by the labs of John Foker and Heinz-Gerd Zimmer, utilised ribose and adenine infusions in various in vitro and in vivo cardiac preparations. These studies were successful in restoring both cardiac ATP levels and cardiac function after damage caused by ischemia or adrenoceptor activation [42–46]. Indeed, such was the interest in this approach that a number of patents were filed in the 1980s [47]. However, the influence of high doses (50 mg/kg) of adenine on the cardiovascular system, its nephrotoxicity when converted to the insoluble 2,8-dihydroxyadenine, and the prolonged (24–48 h) intra-cardiac route of infusion in some studies has not seen a pursuit of this combined approach in humans. Nonetheless, ribose per se is still being considered as an adjunct to cardiac rehabilitation [48, 49], whilst for adenine, early reports of cardiac benefits of an adenine-containing mixture (“Purinor”; [50]) may have led to the continued use of Purinor in some countries.

### ATP Loss and Replenishment in Brain Tissue: Lessons From the Heart

**Fig. 2:**
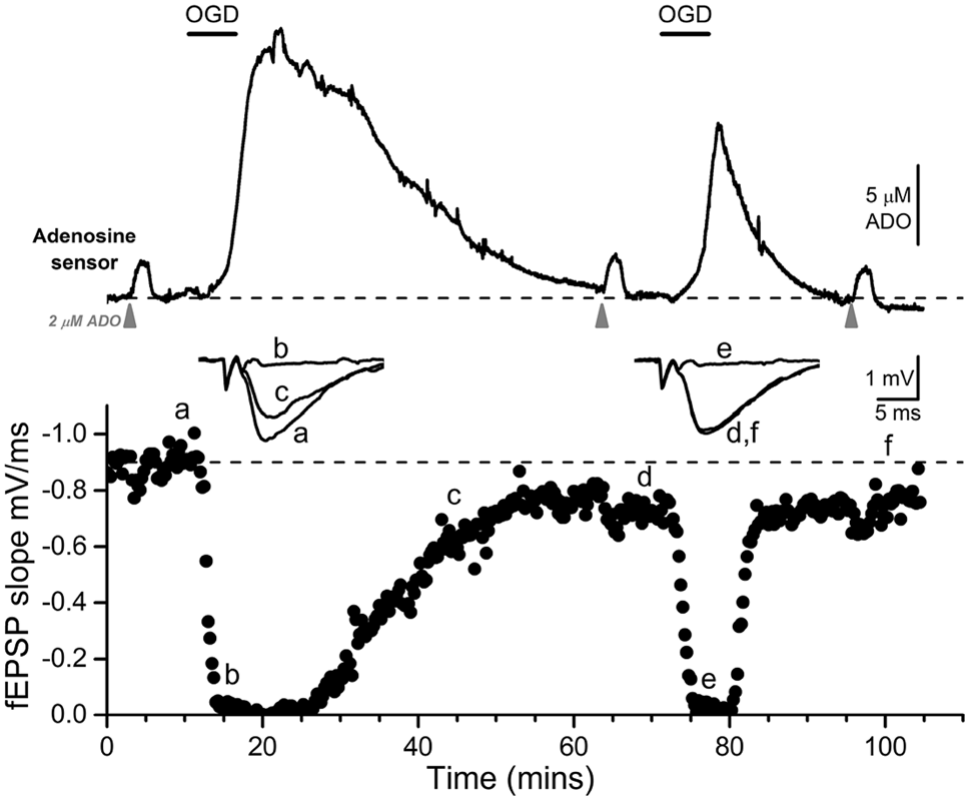
Evidence for adenosine depletion in area CA1 of hippocampal slices. Top panel shows adenosine sensor measurements of adenosine (ADO) release in response to two identical periods of oxygen/glucose deprivation (OGD, 6 min, *black bar*). Lower panel shows the depression and recovery of the simultaneously recorded fEPSP. * Inset* are fEPSPs taken at the times indicated. Note the reduced release of adenosine and reduced effects on the fEPSP during the second period (compare fEPSPs *c* and *f* taken at comparable times after OGD). *Grey triangles* refer to applications of exogenous adenosine (2 µM) to test that the sensor has not run down over this period. In these experiments, differential measurements were made between adenosine and inosine biosensors resulting in net adenosine release being detected. Adapted from [33]

We were initially drawn to the issue of ATP levels in brain slices through observations that the release of adenosine provoked by hypoxia or OGD, which is largely responsible for the inhibition of excitatory synaptic transmission during and after metabolic stress in vitro [51–55] and in vivo [56–58], was reduced the second time the insult was delivered (Fig. 2). This manifested as a delay in the depression and acceleration in the recovery of excitatory synaptic transmission [59]. Whilst this could reflect an artefact of the slice preparation, similar observations of reduced adenosine release were being made around that time in in vivo preparations [60–62]. Furthermore, it was known that such repetitive insults to the brain could provoke greater brain damage [63], a fact that is of great current concern in the context of sport-related concussions [64]. Thus, these observations of ATP and adenosine depletion had potential clinical importance [65].

One possibility was that the reduced adenosine release reflected a depletion of the primary reservoir for adenosine, ATP. This was plausible as the brain, like the heart, relies largely upon the purine salvage pathway for the restoration of adenine nucleotides, as de novo synthesis is slow and not increased after metabolic stress [66–68]. Indeed, that the substrates of the purine salvage pathway (Fig. 1) are depleted in the ischemic brain are dramatically demonstrated by Matrix Assisted Laser Desorption/Ionization (MALDI) imaging [69] of ex vivo ischemic brain tissue. Such studies have revealed the loss of ATP, ADP, AMP, adenosine, inosine, hypoxanthine and ribose-5 phosphate in the ischemic core, but with accumulations of the non-salvageable xanthine and uric acid [70, 71].

Accordingly, the release of adenosine into the perfusate could, as in the heart, represent a loss of substrates for the purine salvage pathway. That the release of adenosine and other purines has been observed in the blood stream in humans experiencing cerebral ischemia [72] lends support to this possibility, and potentially explains the protracted recovery of cerebral bioenergetics after concussion [73]. Moreover, purines in the blood could serve as rapid diagnostics for stroke.

Attempts to reverse adenosine depletion via the provision of exogenous adenosine were successful [59], suggesting that the slice had the capacity to restore its depletable pool of both adenosine and, by inference from the work of Thomas [24], ATP. Given the limitations of adenosine described above, we subsequently adopted the ribose/adenine approach pioneered in the heart.

We initially confirmed observations made on numerous occasions previously eg [74, 75] as to the impoverished level of ATP in hippocampal slices, even when slices are incubated in supra-physiological levels of glucose (10 mM; Fig. 2) [23]. We additionally examined the energy charge (EC) [76] of the slices, which conveys the energetic state of a cell and is given by the following equation:

$$EC=\frac{{[ATP]+0.5[ADP]}}{{\left[ {ATP} \right]+\left[ {ADP} \right]+[AMP]}}$$

such that if all the adenine nucleotides were in the form of ATP energy charge would equal 1, whilst if only AMP was found the energy charge would equal 0. We found that, despite the attenuation in ATP levels, EC was close to values recorded in vivo (typically ~0.95) [23, 77]. This suggests that a new metabolic equilibrium had been achieved amongst the remaining adenine nucleotides. This was only true, however, if care was taken to extract adenine nucleotides from neutralised fresh tissue; freeze-thawed tissue showed EC values similar to those widely reported for brain slices (<0.9) [77].

**Fig. 3:**
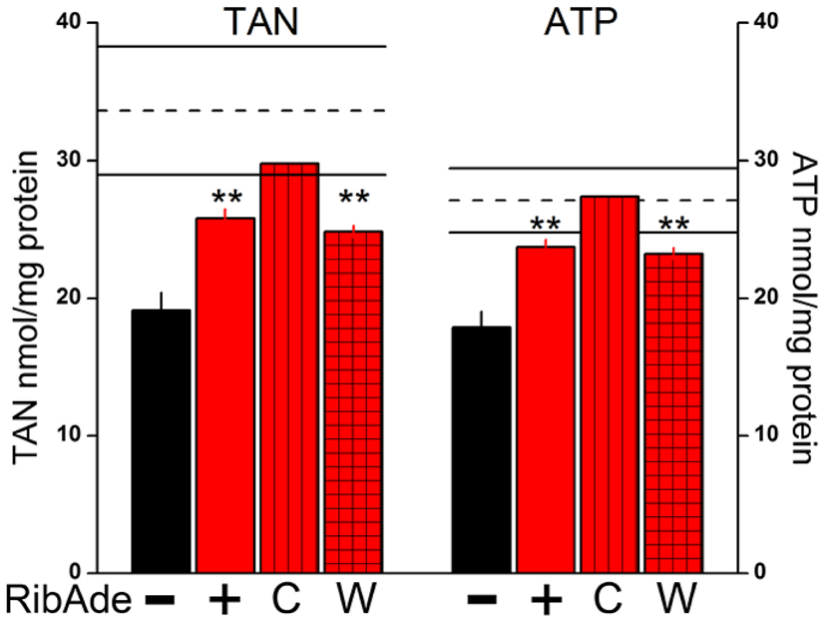
Ribose and adenine (RibAde) elevate brain slice adenine nucleotides. Incubation in RibAde (+; *solid red bar*) increased total adenine nucleotide content (TAN; Σ(ATP + ADP + AMP)) and ATP levels above control levels in hippocampal slices (−; *black bar*). This increase persisted upon washout of RibAde from the incubation medium (W; 2 h; *checked red bar*). When TAN and ATP levels were corrected for the dead edges of slices (C; *vertical hatched red bar*), slice TAN and ATP were within the range reported in vivo (mean, *dashed line; solid lines* ± 1 SD; references found in [23]). **p < 0.01 sig diff from control slices (−). Adapted from [23]. (Color figure online)

Incubation of hippocampal slices (with overlying neocortex), from the time of cutting, in 10 mM glucose-containing aCSF supplemented with modest concentrations of ribose (1 mM) and adenine (50 µM; “RibAde”) resulted in increases in the total adenine nucleotide (TAN) and ATP content after 2–3 h (Fig. 3; [23]). Neither were effective alone, and this seemed to be the optimal concentration of both compounds since increases in either did not improve ATP levels (or the EC) any further. The increase in cellular ATP was stable in that removal of slices from RibAde-containing aCSF to control aCSF still resulted in elevated ATP after at least 2 h. Moreover, compensating for the dead cut edges of slices (which contribute protein, but no ATP to the normalisation to protein content) resulted in estimates of tissue adenine nucleotides that were within the range reported in vivo, with estimates of ATP being within a few percent of that measured from the rapidly fixed brain (Fig. 3). This interesting result, together with the inability of higher concentrations of RibAde to enhance ATP levels further, suggests an optimal level of ATP that may be limited by feedback inhibition on ATP-synthesising enzymes [67].

### The Purine Salvage Pathway as the Vehicle for ATP Replenishment

The most likely route by which this ATP is synthesised following the provision of RibAde is via the purine salvage pathway, the enzymes of which are found in brain and, importantly, are located in the cytosol [67, 78] and provide an energetically-efficient means to resynthesise adenine nucleotides (Fig. 1). Provision of exogenous ribose and its phosphorylation by ribokinase to ribose-5-phosphate bypasses the pentose phosphate pathway and is a more direct route by which the cellular PRPP pool can be increased. Endogenous ribose-1-phosphate derived from inosine can be converted to ribose-5-phosphate by phosphopentomutases [79] and hence PRPP (Fig. 1) and this has been described as the main route by which purines are salvaged [67]. The importance of the PRPP pool stems from the fact that it contributes the phosphoribose moiety to adenine and hypoxanthine to generate AMP and IMP via adenine phosphoribosyltransferase (APRT) and hypoxanthine-guanine phosophoribosyltransferase (HPRT), respectively. Both enzymes are expressed in neurones and astrocytes, with similar levels of APRT, but greater expression of HPRT in neurones [80]; mutations in HPRT are responsible for Lesch-Nyhan disease [81]. Two further steps convert IMP to AMP, which can then be converted to ATP via adenylate kinases [68].

Adenylate kinases are interesting enzymes in that there are nine isoforms, several of which are found in brain and localised to the cytosol [82]. Moreover, most isoforms possess both nucleoside monophosphate (eg AMP→ADP) and nucleoside diphosphate (eg ADP→ATP) kinase activity and are capable of utilising phosphate donors other than ATP. This diversity of action and promiscuity in the phosphate donor ensures that a futile cycle of robbing AT(Peter) to pay AD(Paul) does not ensue, but instead the net result is in the generation of cytosolic ATP. In highly polarised cells, such as neurones, having an extra-mitochondrial source of ATP, including via creatine kinase, is very valuable as mitochondria may be remote from the site of neuronal activity. Recent detailed serial electron microscopic reconstructions of the mature neocortex showed that mitochondria were located in only a small fraction of dendritic spines (3 out of 1425 spines) [83]. This contrasts with the situation in primary neuronal cultures where mitochondria or their protrusions may enter postsynaptic spines in an activity-dependent manner [84].

The cytosolic location of the enzymes involved in purine salvage pathway and the conversion of AMP to ATP (Fig. 1) indicates that the local synthesis/replenishment of ATP can occur at sites of high activity and independently of mitochondria, at least in the short-term, and may permit the “on-demand” nature of synaptic adenosine release which dampens presynaptic excitation [85–88]. This local, extra-mitochondrial, generation of ATP has an important additional advantage in the context of brain injury due to the mitochondrial dysfunction that can follow. Such injury-induced dysfunction of mitochondria (including that incurred after slice preparation [89]) can provoke a host of events including mitochondrial *consumption* of cytosolic ATP, and the liberation of reactive oxygen species and pro-apoptotic factors, all of which would contribute to cellular damage [90–92]. It should also be remembered that the extra-mitochondrial purine salvage pathway and adenosine kinase are the mechanisms by which the adenine nucleotide backbone is constructed in the form of AMP. Mitochondria absolutely rely upon this to import cytosolic ADP and generate ATP via oxidative phosphorylation or mitochondrial adenylate kinases. This may explain the limited success of strategies designed to promote ATP synthesis via provision of TCA precursors or intermediates such as lactate or pyruvate [93], or ketones [94] in the context of acute brain injury. These substances do not generally elevate brain slice ATP levels, [95–97], and nor indeed does creatine [98], likely because the adenine nucleotide backbone is not available.

### LTP in ATP-Replenished Brain Slices: Not What You’d Expect

**Fig. 4:**
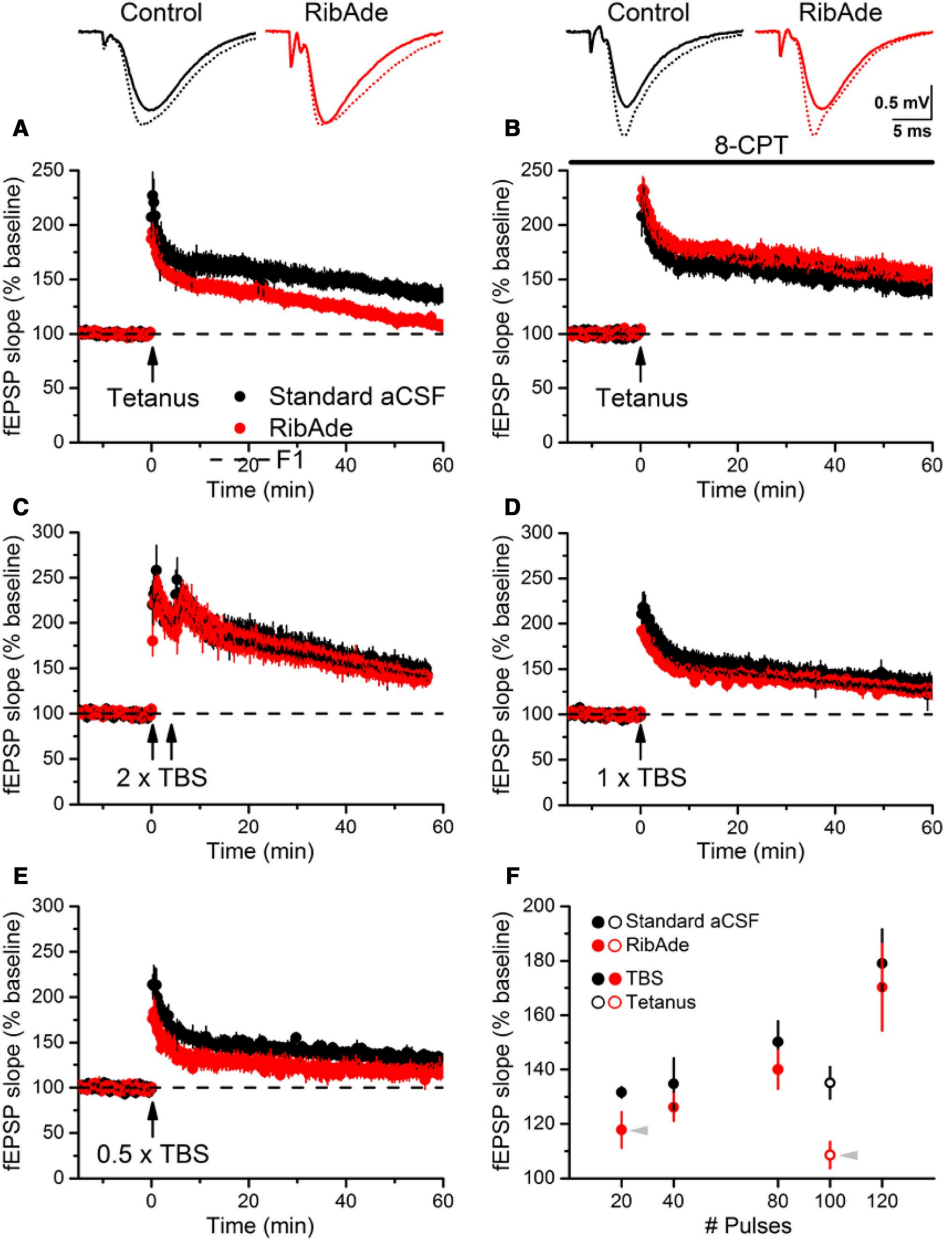
Elevating cellular ATP with RibAde inhibits LTP via adenosine A_1_Rs. **A** LTP induced by a tetanus (100 Hz/1 s; *arrow*) resulted in robust LTP in standard aCSF, but steadily decremented to baseline in slices pre-incubated in RibAde. **B** This decrementing LTP was prevented by the adenosine A_1_R antagonist 8-CPT (1 µM). *Inset* are fEPSPs taken before (*solid lines*) and at 60 min after the induction of LTP (*dashed lines*) in standard aCSF (control) and RibAde-treated slices. **C**–**E** Decreasing the number of TBS pulses (80, 40, 20, respectively) induced LTP, which is consistently lower in RibAde-treated slices. 20 TBS pulses (0.5 x TBS; **E**) failed to induce significant LTP above baseline. **F** Summary of LTP at 60 min induced in (**A**) (tetanus; open symbols), **C**–**E** Data for 120 pulses from the LTP (at 30 min) induced by 3 x TBS used to evoke the adenosine release in Fig. 5A. *Grey arrowheads* indicate where LTP was not significantly greater than baseline in RibAde-treated slices. A two-way ANOVA (standard aCSF or RibAde treatment vs number of pulses) showed a significant effect of treatment on the LTP evoked by the various stimulation protocols (F_1,51_ = 6.47688; p = 0.014). Adapted from [23]

Having now produced slices with physiological levels of cellular ATP, an obvious question was to determine the influence that this would have on long-term potentiation (LTP). Our expectation was that creating such “healthy” slices, LTP would be enhanced or at least facilitated. In contrast, despite there being no effect on basal synaptic transmission or paired-pulse facilitation of RibAde treatment, LTP in response to a 1 s tetanus was inhibited (Fig. 4A). We were able to establish, using an adenosine A_1_ receptor (A_1_R) antagonist that this was due to greater activity-dependent release of adenosine and activation of inhibitory adenosine A_1_ receptors in RibAde-treated slices (Fig. 4B). To establish whether this inhibition of LTP was specific to tetanus-induced LTP, we performed theta-burst stimulation (TBS; Fig. 4C–E) LTP using multiples (2, 1, 0.5, respectively) of a 40 pulse TBS protocol [23]. This induced LTP that was consistently smaller in RibAde-treated slices (Fig. 4F). Indeed, LTP was not significantly induced in RibAde-treated slices given 20 pulses (0.5 x TBS; Fig. 4E).

**Fig. 5:**
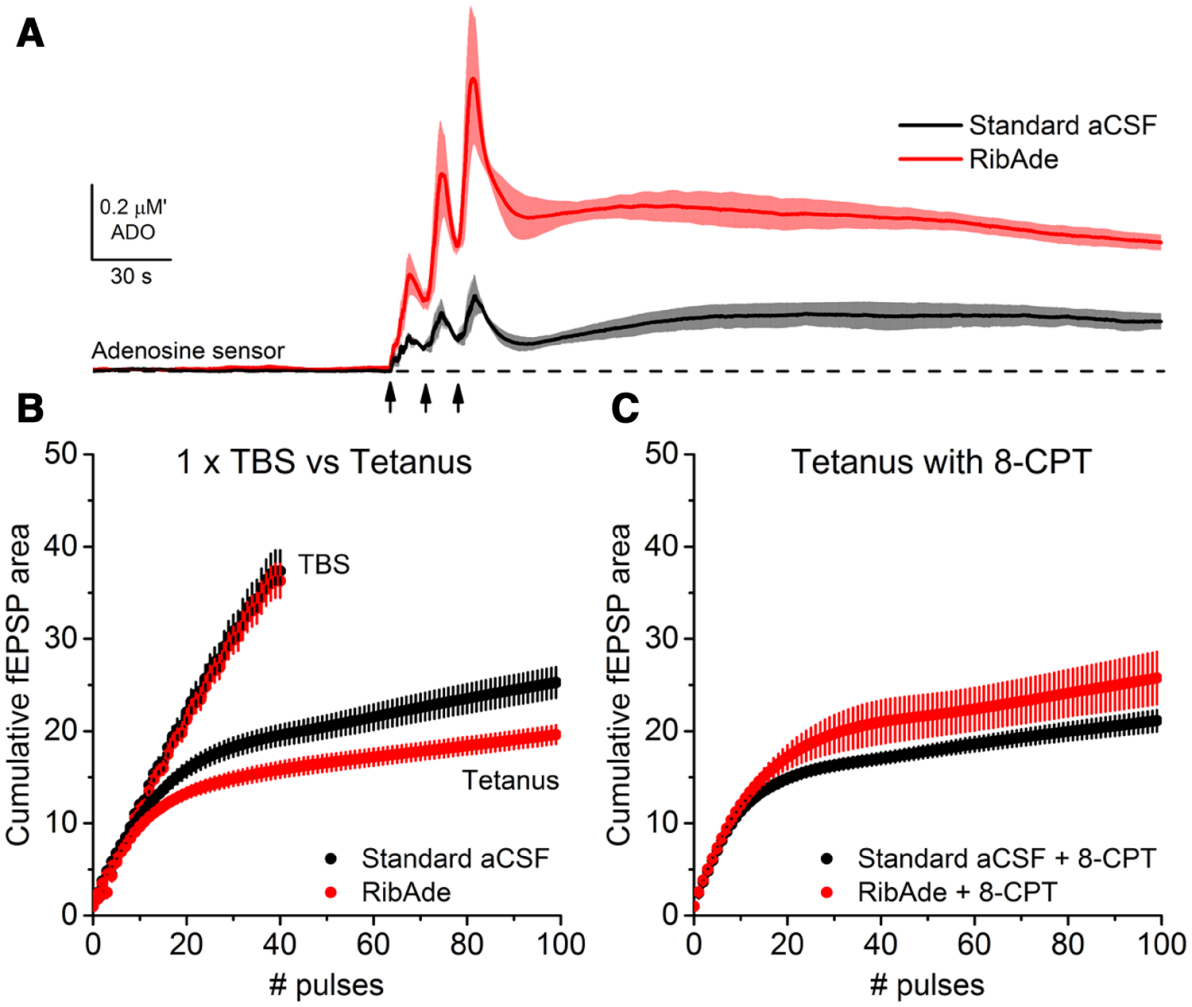
Adenosine release during high-frequency stimulation. **A** RibAde increased adenosine release (ADO) in response to TBS (*arrows*). Note the release per individual theta burst (10 trains at 5 Hz of 4 pulses at 100 Hz) and the sustained post-TBS elevation of adenosine, which may be responsible for the subsequent inhibition of LTP seen in Fig. 4A. In these experiments the biosensor measures the signal recorded from an adenosine sensor, which is sensitive to adenosine and its metabolites (inosine, hypoxanthine and xanthine). The concentration is thus given as µM′ to indicate that it is not purely adenosine that is detected. **B** The envelope of depolarisation evoked by tetanic stimulation fatigues after ~20 pulses, an effect that was enhanced by RibAde. This is likely via increased adenosine release since (**C**) this effect was reversed by the A_1_R antagonist 8-CPT. In contrast, the cumulative depolarisation evoked by TBS (**B**) was unaffected by RibAde, suggesting that spacing between bursts (200 ms) was sufficient to allow lowering of synaptic adenosine to the point that it does not interfere with synaptic transmission, at least during the period of high-frequency stimulation. Adapted from [23]

We surmised that the greater cellular ATP pool had provided for a larger releasable pool of adenosine. Measurements using adenosine biosensors confirmed this supposition (Fig. 5A). That this adenosine was released as such, and not as ATP, was suggested by the lack of effect of the ecto-nucleotidase inhibitor POM-1 [99] on evoked adenosine release. That neuronal activity caused by electrical stimulation results in the breakdown of cellular high-energy phosphates has been known for quite some time [2], as has the fact that this then results in the release of adenosine [100].

An examination of the fEPSP depolarising envelope evoked by 100 Hz stimulation revealed a fatigue of the fEPSP after approximately 20 pulses, which was exaggerated in RibAde-treated slices (Fig. 5B) and which indeed was the number of pulses that failed to induce TBS LTP in RibAde-treated slices (Fig. 4E). This RibAde-induced enhancement of fatigue, as per the inhibition of tetanus-LTP (Fig. 4B), reversed upon treatment with the A_1_R antagonist 8-CPT (Fig. 5C). During TBS the depolarising envelope in response to 40 pulses (1 x TBS), in contrast to the situation with the tetanus, showed was no such fatigue of the fEPSP, and indeed no obvious effect of RibAde. This implies that, in addition to the two properties of TBS that makes it effective in inducing LTP, maximising both postsynaptic depolarisation (by preventing depolarisation block and transmitter depletion) and fatigue of inhibitory GABAergic transmission, we may potentially add a third—preventing the synaptic accumulation of adenosine during synaptic activity. However, since LTP *was* reduced by RibAde, some inhibitory action of adenosine must occur post-induction, which is reminiscent of observations made by Gary Lynch in the 1990s regarding an early phase of LTP sensitive to adenosine [101]. Direct measurements of adenosine release in response to TBS demonstrates the persistence of adenosine in the extracellular space after the induction of LTP during the early sensitive period (Fig. 5A).

### ATP and OGD

**Fig. 6:**
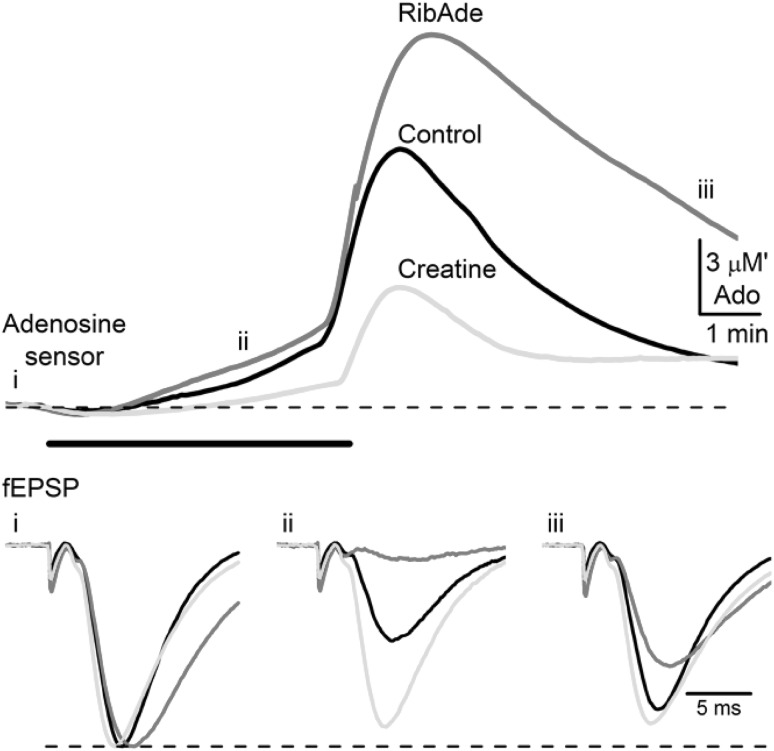
Modulation of intracellular ATP influences adenosine release and synaptic transmission during metabolic stress. *Upper panel* shows adenosine (ADO) release in response to a 5 min period of oxygen/glucose deprivation (OGD, *black bar*) in control slices, and slices pre-incubated in RibAde and creatine. Note greater release of adenosine in RibAde-treated slices and reduced release in slices pre-incubated in creatine (mean of 4 traces for each condition shown). Lower traces show fEPSPs, taken at the times indicated and colour-coded as per adenosine release, demonstrating the (*ii*) accelerated depression and (*iii*) delayed recovery of synaptic transmission in RibAde-treated slices compared to the delayed depression and accelerated recovery in creatine-treated slices. These observations are in keeping with the enhanced adenosine release caused by RibAde, and the reduced adenosine release caused by creatine. fEPSPs have been normalised to the pre-OGD amplitude, which is indicated by the *dashed line*. Adapted from [98]

Given that our interest in the fate of ATP and adenosine arose from work conducted in the context of OGD, we returned to this issue to examine the effects of metabolic stress in slices preincubated in RibAde. We predicted that the enhanced levels of ATP should result in greater adenosine-mediated inhibitory actions on excitatory synaptic transmission. As a comparator, we performed additional studies using creatine (1 mM). By buffering the decline in cellular ATP through the creatine kinase-mediated phosphotransfer from phosphocreatine to ADP, ATP levels are preserved, and should result in a delay and reduction in the appearance of extracellular adenosine, with subsequent reduced inhibitory effects on excitatory synaptic transmission. These predictions were borne out [98] (Fig. 6) and furthermore demonstrated that previous observations [102] concerning the sparing of synaptic transmission and neuronal excitability by creatine were not entirely due to better ATP-dependent support for neurotransmitter release per se, but by reduced extracellular adenosine release. Similar observations of RibAde and creatine facilitating and reducing adenosine release during seizure activity, with inhibitory and excitatory actions on neuronal activity, respectively, have been made in an in vitro model of epileptiform activity [103]. This suggests that the seizure-induced depletion of intracellular ATP contributes to the rapid accumulation of extracellular adenosine to limit the duration, frequency and intensity of seizures [33].

### Therapeutic Potential of RibAde-Based Therapy

Given the ability of RibAde to: elevate ATP levels in the injured brain (slice); increase the release of neuroprotective adenosine in response to physiological and pathological stimuli; exert an adenosine A_1_R-dependent inhibitory influence on excitatory synaptic transmission, and indeed to protect cerebellar granule cells when administered after OGD [98], to what extent might RibAde-based therapy be of value in the injured human brain? This would depend upon two key factors, namely (1) the safety and tolerability of ribose and adenine, and (2) the extent to which they can cross the blood–brain barrier from the systemic circulation.

#### Safety and Tolerability

On the former, ribose is widely sold as an unregulated nutritional supplement, and large quantities can be ingested orally with few side effects [104]. In terms of its intravenous delivery, escalating doses of ribose were administered intravenously for 12 h in humans until 220 mg/kg was administered for 4 h [105]; this dose is higher than that used by Zimmer in animal models (200 mg/kg). The study by Gross and Zöllner was designed to investigate the mild blood glucose-lowering ability of ribose (20–25%), which has been known since the 1940s. However, in the context of the injured brain, glucose-lowering therapies may mitigate against the damage caused by injury-induced hyperglycaemia [106] and may thus be an additional benefit of ribose-based therapy.

Adenine is used to support red blood cell ATP production in blood transfusion products. Red blood cells lack both mitochondria and de novo synthesis of adenine nucleotides and thus rely on the purine salvage pathway. Inclusion of adenine in red blood cell transfusion products doubled the shelf-life of fresh blood from 3 to 6 weeks, whilst some experimental adenine-containing solutions can extend viability to 8–10 weeks [107]. High doses of adenine, however, result in toxicity and pathology associated with the xanthine oxidase-mediated conversion of adenine to the insoluble 2,8 dihydroxyadenine, which forms crystals, primarily in the kidney. This occurs in APRT deficiency, but can be managed via the use of the xanthine oxidase inhibitor, allopurinol [108]. Given this concern, the introduction of adenine as a component of blood transfusion products was delayed in some countries until extensive animal and human studies established safe limits for intravenous infusion, which are in the region of 10–15 mg/kg [109]. However, simulations of massive blood transfusions at that time indicated that higher doses (up to 100 mg/kg) could be tolerated in non-human primates [110], and indeed there appears to be only one report of adenine-based kidney damage in humans having received massive blood transfusions subsequent to major trauma or obstetric haemorrhage [111]. Thus, whilst there are potential issues associated with adenine, the infusion over an extended period, the spontaneous deamination of adenine to hypoxanthine, together with the inclusion of allopurinol to inhibit its conversion to 2,8 dihydroxyadenine, would mitigate against the toxicity of adenine.

In this regard, allopurinol may have benefits additional to preventing the conversion of adenine to 2,8 dihydroxyadenine; by similarly preventing the conversion of hypoxanthine to the non-salvageable xanthine (Fig. 1), allopurinol would simultaneously allow greater availability of endogenous hypoxanthine for the salvage pathway. Sparing of purines during cerebral ischemia by allopurinol or the active metabolite oxypurinol has indeed been observed, with functional benefits [112, 113]. In addition to purine-sparing actions of xanthine oxidase inhibition, such inhibitors would also prevent the production of reactive oxygen species created by the subsequent metabolism of xanthine which yields hydrogen peroxide. This double benefit of allopurinol is being tested in the context of the hypoxic/ischemic neonate to whom allopurinol can be administered via the maternal/placental circulation [114].

#### Penetration into the Brain

Since ribose, adenine and indeed allopurinol (eg for the treatment of gout) are all in use in man, to what extent might they enter the brain via the systemic circulation? Ribose is a peculiar compound in that it is extremely permeable through lipid membranes, a fact that may have permitted the emergence of the ribose-based RNA world [115, 116]. Its rapid uptake into brain was demonstrated following tracking of radiolabelled ribose [117]. In contrast to the liver [117, 118], ribose taken up into the brain is not converted to glucose [117]. Whether its uptake occurs by diffusion [119, 120] or via a transport mechanism [121] is not clear. Uptake may occur via a glucose transporter since it partially interferes with glucose transport [122], potentially GLUT2 [118], which is found in brain [123]. Indeed other members of the SLC2 glucose transporter family may, in addition to glucose, transport pentose sugars [124, 125].

The evidence for adenine uptake into the brain is clearer [126] and likely occurs via the equilibrative nucleoside transporters (ENTs; SLC29). ENT1 and ENT2 have a fairly ubiquitous cell surface expression and are capable of mediating adenine (and hypoxanthine) transport across the blood brain barrier [126–129]. It is also likely that allopurinol enters the brain in this way, given its closely related structure to hypoxanthine (Fig. 1), and indeed the uptake of adenine, hypoxanthine and allopurinol interfere with each other [130]. In contrast, access to the brain of creatine is severely limited by the absence of creatine transporters on the astrocytic endfeet that envelop cerebral blood vessels and thereby requiring extensive pre-treatment to demonstrate benefits in animal models of brain injury [131].

### Therapeutic Potential of RibAde-Based Therapy: The Proof of Concept is in the Stroke Model

**Fig. 7:**
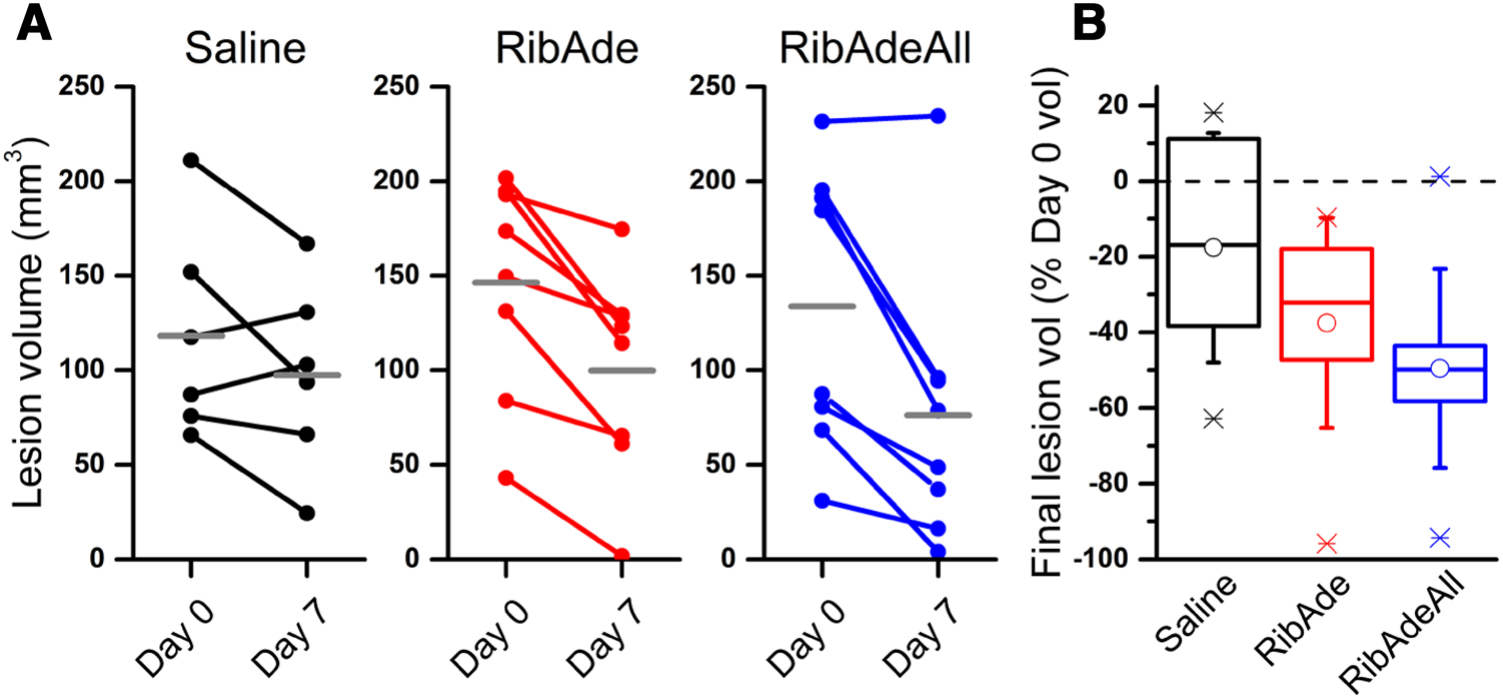
RibAde and RibAdeAll (RibAde plus allopurinol) showed a trend towards reduced lesion volume in a pre-clinical model of stroke. **A** Individual paired measurements of lesion volume at Day 0 and Day 7 in the three experimental groups of animals showing a consistent trend for substantial lesion shrinkage in the RibAde (n = 8) and RibAdeAll (n = 8) groups compared to saline controls (n = 6). *Grey horizontal bars* show mean lesion volume by day. **B** Change in lesion volume between Days 0 and 7 normalized to lesion size on Day 0. Mean lesion size (*open circles*) decreased by 18% in the saline group, 38% in the RibAde group and 50% in the RibAdeAll group. Horizontal line, median; box 25–75% range; whiskers, ±1SD; X, min and max values. Adapted from [132]

**Fig. 8:**
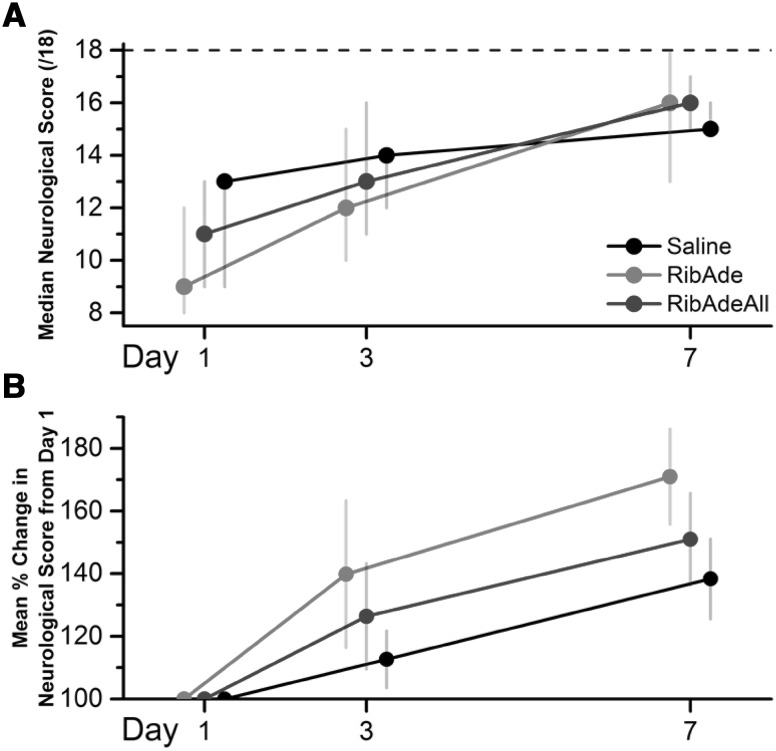
RibAde and RibAdeAll treatment resulted in a tendency towards improved neurological outcome after stroke. All animals scored the maximum value of 18 prior to transient middle cerebral artery occlusion (tMCAO; *dashed line* on **A**). **A** Median neurological scores (from minimum of 3 to maximum of 18; *dashed line*) per group on Day 1, 3 and 7 after induction of stroke (n = 8 in RibAde and RibAdeAll groups and n = 6 in saline group). *Bars* indicate the 1st and 3rd quartiles for each data point. Median and quartile values are rounded to nearest whole number. Data points for each group have been offset for clarity. **B** Mean neurological scores (±SEM) for each day normalised to Day 1 revealed a trend to faster recovery despite larger initial lesions in the treatment groups, consistent with the greater reduction in lesion size (Fig. 7). Data points for each group have been offset for clarity. Adapted from [132]

These considerations were recently put the test in an in vivo model of stroke. Rats underwent a transient, 60 min, filament occlusion of the middle cerebral artery and, upon reperfusion, were administered an intravenous 6 h infusion of either saline, RibAde (200 and 10 mg/kg, respectively) or RibAde plus an intraperitoneal injection of the xanthine oxidase inhibitor, allopurinol, (10 mg/kg; RibAdeAll). MRI scans were taken prior to infusion and at day 7. Neurological testing occurred at days 1, 3 and 7. RibAde- and RibAdeAll-treated animals displayed a strong tendency towards reduced lesion volumes compared to saline-treated rats (Fig. 7), and evidence of accelerated neurological recovery (Fig. 8). This study [132] was underpowered and did not achieve statistical significance, but does provide valuable proof of concept information in that these doses of ribose, adenine and allopurinol are tolerated, do not cause kidney damage and may indeed reduce brain damage and improve functional recovery after injury. Subsequent studies would need to be adequately powered (we estimate 13 animals per group) and, as per all preclinical stroke work, should adhere to appropriate STAIR [133], ARRIVE [134] and the recent IMPROVE [135] guidelines.^1^

## Conclusions

The ATP content of brain slices can be restored to values recorded in vivo. This has immediate consequences in increasing the cellular reservoir of the neuromodulator adenosine with, as we have shown, quite pronounced effeicts on synaptic transmission and plasticity under both physiological and pathological conditions. Wider adoption of RibAde in slice incubation media might reveal other effects of increasing tissue ATP levels, for example in an enhancement in the size of synaptic events mediated by the release of ATP and activation of ionotropic P2X receptors, of greater modulatory actions of GPCR P2Y receptors, and indeed on adenosine receptors beyond the inhibitory A_1_ receptors whose actions we have studied. Cellular biochemistry may also be influenced in terms of appropriate utilization of cellular energy sources, the balance between glycolysis and oxidative phosphorylation, and the kinetics and extent of cellular reactions, such as phosphorylation, that require ATP.

In terms of what RibAde-based approaches may mean for the injured brain remains to be seen, but the pilot data in the preclinical stroke model are encouraging. The loss of substrates for the purine salvage pathway after the cerebral ischemia and trauma associated with brain slice preparation has strong parallels with what happens during these conditions in humans and experimental animals. Accordingly, post-injury infusion of RibAde may be of value in restoring cerebral ATP levels in tissue peripheral to the injury, but vulnerable to expansion of the damaged area. This penumbral tissue is potentially salvageable and the focus of existing therapies such as thrombectomy or thrombolysis for ischemic stroke, and the lowering of intracranial pressure after traumatic brain injury. Restoring ATP in these regions would: (1) facilitate better maintenance of membrane potential in the face of depolarising waves (spreading depression/depolarizations, peri-infarct depolarisations) commonly associated with brain injury; (2) allow cells to activate reparative mechanisms, such as protein synthesis, which is inhibited after injury, and (3) promote the release of greater amounts of adenosine with its inhibitory, anticonvulsant influence on neuronal activity, and its vasodilatory actions on the cerebral vasculature. These actions of adenosine may mitigate against the seizure activity frequently seen after brain injury, and may prevent entry into an epileptogenic state, a common sequela of severe brain injury; improved blood flow will deliver oxygen and glucose to help protect and repair vulnerable brain tissue.

As speed is of the essence in the treatment of brain injury, one could imagine civilian or military paramedics being equipped with saline packs containing RibAde, with for example, allopurinol (RibAdeAll), in order that treatment may be initiated at the point of injury. Given the increase in both civilian and military head injury victims, who are generally in mid-life or younger, and the ageing population, with the associated risk of stroke, such an approach may be of value in reducing the consequences of the brain injury and the individual and familial suffering that it brings, not to mention the huge societal costs of brain injury in terms of treatment, care and lost earnings.

## Footnotes

1 STAIR—Stroke Therapy Academic Industry Roundtable; ARRIVE—Animal Research: Reporting of In Vivo Experiments; IMPROVE —Ischaemia Models: Procedural Refinements Of in Vivo Experiments.
